# Entomological characterization of *Aedes* mosquitoes and arbovirus detection in Ibagué, a Colombian city with co-circulation of Zika, dengue and chikungunya viruses

**DOI:** 10.1186/s13071-021-04908-x

**Published:** 2021-09-06

**Authors:** María C. Carrasquilla, Mario I. Ortiz, Cielo León, Silvia Rondón, Manisha A. Kulkarni, Benoit Talbot, Beate Sander, Heriberto Vásquez, Juan M. Cordovez, Camila González, RADAM-LAC Research Team, Beate Sander, Manisha A. Kulkarni, Jianhong Wu, Camila González, Marcos Miretti, Mauricio Espinel, Varsovia Cevallos

**Affiliations:** 1 Centro de Investigaciones en Microbiología y Parasitología Tropical (CIMPAT), Universidad de Los Andes, Bogotá, Colombia; 2 School of Epidemiology and Public Health, University of Ottawa, Ottawa, Canada; 3 Toronto General Hospital Research Institute, University Health Network, Toronto, Canada; 4 Institute of Health Policy, Management and Evaluation, University of Toronto, Toronto, Canada; 5 Public Health Ontario, Toronto, Canada; 6 Institute for Clinical Evaluative Sciences (ICES), Toronto, Canada; 7 Secretaría de Salud de Ibagué, Ibagué, Colombia; 8 Grupo de Investigación en Biología Matemática y Computacional (BIOMAC), Universidad de Los Andes, Bogotá, Colombia

**Keywords:** Dengue, Zika, Chikungunya, *Aedes aegypti*, *Aedes albopictus*, Colombia

## Abstract

**Background:**

Dengue, Zika and chikungunya are arboviruses of significant public health importance that are transmitted by *Aedes aegypti* and *Aedes albopictus* mosquitoes. In Colombia, where dengue is hyperendemic, and where chikungunya and Zika were introduced in the last decade, more than half of the population lives in areas at risk. The objective of this study was to characterize *Aedes* spp. vectors and study their natural infection with dengue, Zika and chikungunya in Ibagué, a Colombian city and capital of the department of Tolima, with case reports of simultaneous circulation of these three arboviruses.

**Methods:**

Mosquito collections were carried out monthly between June 2018 and May 2019 in neighborhoods with different levels of socioeconomic status. We used the non-parametric Friedman, Mann–Whitney and Kruskal–Wallis tests to compare mosquito density distributions. We applied logistic regression analyses to identify associations between mosquito density and absence/presence of breeding sites, and the Spearman correlation coefficient to analyze the possible relationship between climatic variables and mosquito density.

**Results:**

We collected *Ae. aegypti* in all sampled neighborhoods and found for the first time *Ae. albopictus* in the city of Ibagué. A greater abundance of mosquitoes was collected in neighborhoods displaying low compared to high socioeconomic status as well as in the intradomicile compared to the peridomestic space. Female mosquitoes predominated over males, and most of the test females had fed on human blood. In total, four *Ae. aegypti* pools (3%) were positive for dengue virus (serotype 1) and one pool for chikungunya virus (0.8%). Interestingly, infected females were only collected in neighborhoods of low socioeconomic status, and mostly in the intradomicile space.

**Conclusions:**

We confirmed the co-circulation of dengue (serotype 1) and chikungunya viruses in the *Ae. aegypti* population in Ibagué. However, Zika virus was not detected in any mosquito sample, 3 years after its introduction into the country. The positivity for dengue and chikungunya viruses, predominance of mosquitoes in the intradomicile space and the high proportion of females fed on humans highlight the high risk for arbovirus transmission in Ibagué, but may also provide an opportunity for establishing effective control strategies.

**Graphical abstract:**

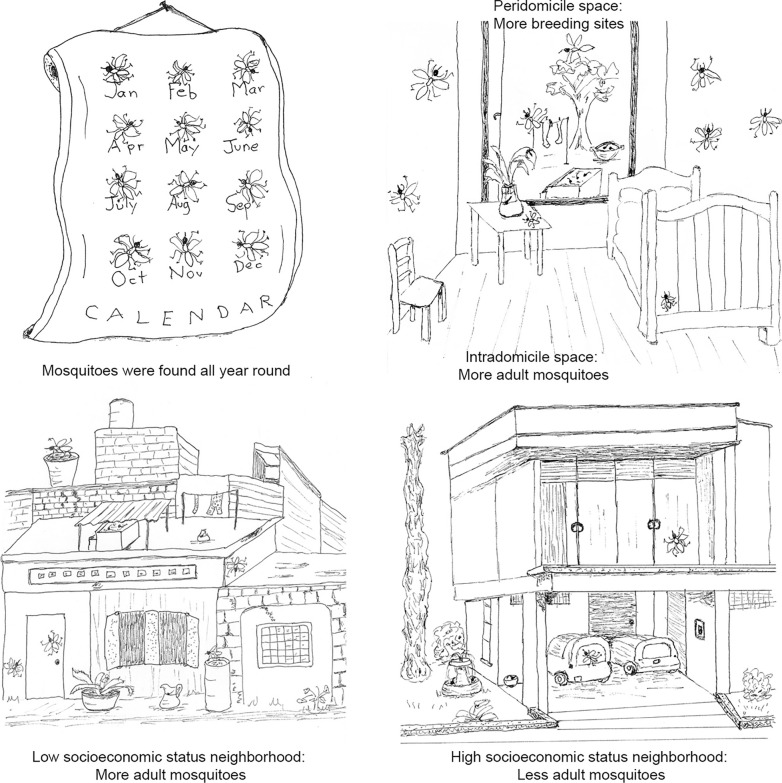

**Supplementary Information:**

The online version contains supplementary material available at 10.1186/s13071-021-04908-x.

## Background

Dengue virus (DENV), Zika virus (ZIKV) and chikungunya virus (CHIKV) are all arboviruses with high public health impact. The incidence of dengue fever has increased substantially over the last two decades worldwide [1], and it is estimated that approximately 390 million dengue infections occur every year [2]. In the Americas, DENV is endemic, whereas CHIKV and ZIKV were introduced in the last decade.

CHIKV was first identified in Tanzania, East Africa, in 1952, and only sporadic cases and a few outbreaks were reported until the early 2000s in Africa and Asia [3]. Between 2007 and 2014, local transmission occurred for the first time in regions with no previous CHIKV local transmission, including the Americas [4]. Notably, in December 2013, autochthonous transmission of CHIKV was detected in the Americas and by March 2015, more than 1.28 million suspected cases, 26,000 confirmed cases and 184 associated deaths had been reported as the virus spread throughout more than 50 countries and territories in the region [4]. ZIKV was identified in Uganda in 1947 [5] and was detected in the Americas in 2015, shortly after the introduction of CHIKV. Before 2007, circulation of ZIKV was limited to parts of Southeast Asia and tropical Africa, where only few cases and disease manifestations had been reported [6]. Between 2015 and 2016, ZIKV spread to 48 countries and territories in the Americas, with approximately 1.5 million confirmed and suspected cases [7]. In some countries, including Brazil and Colombia, local transmission continues to be reported, although with a decline in the number of cases after the epidemic period [7–9].

In Colombia, approximately 28 million people, corresponding to more than half of the national population, is at risk of acquiring these arboviruses [10]. In this country, dengue fever (also dengue) is an endemoepidemic disease, with peaks of DENV transmission every 3–5 years [11, 12]; between 2014 and 2019 around 504,414 dengue fever cases were reported [13]. CHIKV and ZIKV were detected for the first time in the country in 2014 and 2015, respectively [14, 15], and since their introduction until 2019, 488,378 cases of chikungunya and 109,995 cases of Zika virus disease (Zika) have been notified [8, 16–20]. The majority of dengue, Zika and chikungunya cases in the country are reported in only a few departments, such as Tolima [8, 17, 18]. Between 2014 and 2019, 45,325 dengue, 44,570 chikungunya and 7,392 Zika cases were reported in Tolima [8, 13, 16–20]. In Ibagué, a medium-sized city and the capital of Tolima, DENV, ZIKV and CHIKV circulate simultaneously, based on national epidemiological surveillance reports [13]. In this city, DENV circulates throughout the entire year with distinct epidemic periods, while CHIKV and ZIKV showed epidemic transmission after their introduction, followed by a decreasing trend in the number of reported cases [13].

DENV, ZIKV and CHIKV are mainly transmitted by *Ae. aegypti* and *Ae. albopictus *mosquitoes. In Colombia, *Ae. aegypti* is widespread in populated areas located below 2200 m.a.s.l. [21]. *Aedes albopictus* was first found in the country in 1998 [22], and since then has been reported in 15 of 32 Colombian departments [23–27].

In Colombia, efforts have been made to reduce dengue incidence, including integrated vector control, entomological and epidemiological surveillance and clinical case management. However, widespread transmission of DENV remains persistent. The introduction of CHIKV and ZIKV in the last decade has made the situation even more complex [10, 12]. In areas where DENV, ZIKV and CHIKV circulate simultaneously, entomological research is of particular interest as few studies have identified virus co-circulation in field-caught mosquitoes [28, 29]. In the present study, our objective was to perform an entomological characterization of *Aedes* spp. populations in Ibagué, Colombia and to study their natural infection with DENV, ZIKV and CHIKV. Entomological characterization was performed through the assessment of multiple factors relevant to arboviral transmission, including household socioeconomic condition, mosquito and virus seasonality, infection rates and feeding preferences.

## Methods

### Study site

Our study was carried out in Ibagué, the capital and largest city of the Tolima department, in Colombia. The city has 450,785 inhabitants, occupies an area of 1498 km^2^, is situated at an average elevation of 1225 m.a.s.l. and has an average temperature of 24 °C [30]. We selected this study site based on the high incidence of dengue, Zika and chikungunya [13].

### Mosquito sampling

Sampling was performed monthly between June 2018 and May 2019 with the support of Ibagué’s Health Department. Neighborhoods in Comuna 9 in Ibagué were classified by high/low socioeconomic status (SES) according to Colombian stratification. The classification of socioeconomic strata (referred as “estrato”) is based on housing and environmental characteristics and defines the manner in which fees for household utilities are charged. The Colombian government has defined six socioeconomic strata. Strata 1, 2 and 3 correspond to low socioeconomic strata, in which utilities are subsidized, while households classified in strata 5 and 6 have to pay an extra fee. Households in stratum 4 are not subsidized and do not pay additional costs [31].

**Fig. 1:**
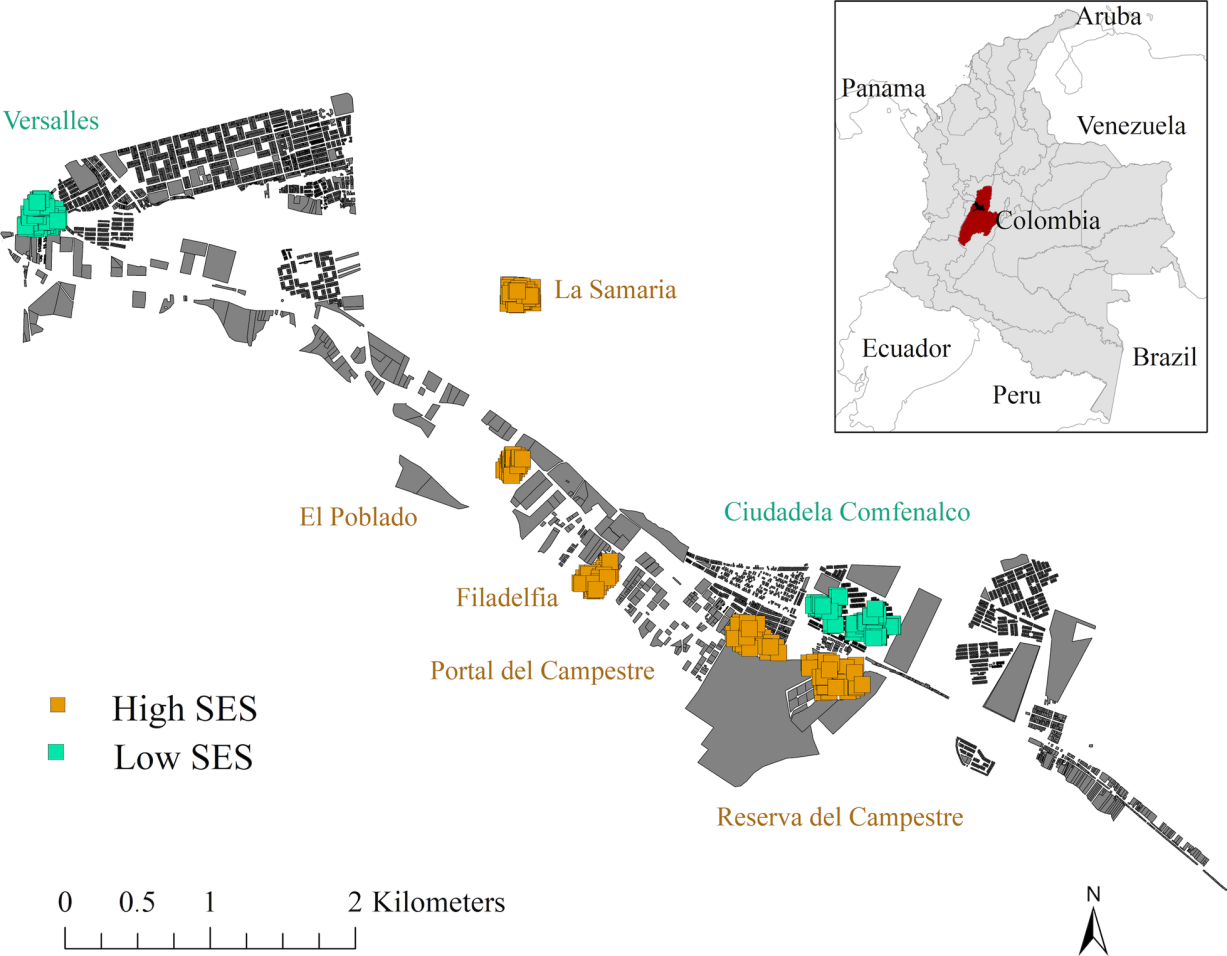
Sampled neighborhoods in Comuna 9, Ibagué, Tolima, Colombia. SES Socioeconomic status

Four neighborhoods were randomly selected, with two in each SES stratum, for mosquito collection. Every month, two high (stratum 5 and 6) and two low (stratum 2) SES neighborhoods were sampled and in each neighborhood eight different houses were inspected, except for 1 month, in which only six houses were sampled in one of the neighborhoods, corresponding to a total of 96 households per neighborhood. In high SES neighborhoods it was not possible to obtain the desired number of households (96), so additional randomly selected high SES neighborhoods were inspected during the course of the study to achieve the target sample size (Fig. 1).

### Adult mosquito collection

Mosquito collections were performed in 382 houses. In each dwelling, mosquitoes were captured using Prokopack aspirators (John W. Hock Co., Gainesville, FL, USA), for 30 min in the intradomicile space and 30 min in the peridomicile space. In addition, a BG-Sentinel trap (Biogents AG, Regensburg, Germany) using a BG-lure was installed in the intradomicile space, while a CDC miniature light trap (John W. Hock Co.) and a resting trap (BioQuip Products, Rancho Dominguez, CA, USA) were set up in the peridomicile area for 24 h. The intradomicile space corresponded to enclosed areas inside the house, such as bedrooms, living room, dining room, bathrooms and kitchen, while the peridomicile space consisted of open areas of the property, such as patios, gardens, porches and roof terraces.

In each house, a household questionnaire was administered and meteorological data (temperature and relative humidity) were registered using an I-button (iButton® Hygrochron logger; Maxim Integrated, San José, CA, USA). Precipitation data were obtained from the Instituto de Hidrología, Meteorología y Estudios Ambientales [32]. In the field, mosquitoes were anesthetized with ethyl acetate, and using a dissecting microscope, the species *Ae. aegypti* and *Ae. albopictus* were identified [33]; sex and female feeding status (blood fed or non-blood fed) was determined as described elsewhere [34]. Mosquitoes were kept in RNAlater at 4 °C and in the laboratory they were frozen at – 80 °C in a Revco freezer (Thermo Fisher Scientific, Waltham, MA, USA).

### Collection of immature mosquitoes

An active search was made of mosquito breeding sites in the selected dwellings. Containers that store water in the intradomicile and peridomicile spaces of each house were inspected to determine the presence of immature mosquito stages. Using a “turkey baster” pipette, a sample of approximately 500 ml was taken, and immatures were identified [33, 35].

### Statistical analysis

A weighted average for 24-h temperature and humidity was calculated for each house. Kolmogorov–Smirnov and Shapiro–Wilk tests were used to examine data distribution. Friedman and Mann–Whitney tests were used to compare mosquito density values. Logistic regression analyses were used to investigate associations between entomological indices, including adult mosquito abundance and presence of breeding sites, and meteorological conditions, including temperature, humidity and precipitation. Kruskal–Wallis tests were used to compare monthly mosquito abundance. The Spearman correlation coefficient was applied to establish relationships between mosquito abundance and temperature, humidity and precipitation. The Mann–Whitney U-test and Fisher’s exact test, corrected using Bonferroni’s method, were applied to characterize variations in household characteristics. Also, Fisher’s exact test was used to compare breeding sites found in the intradomicile versus breeding sites found in the peridomicile. The program Real-Statistics for Excel 7.2 (Microsoft Corp., Redmond, WA, USA) was used.

### Molecular extraction

Female mosquitoes were sorted by species, house and location of the traps (intradomicile or peridomicile space). Samples collected in the same house, and consequently on same sampling date, were pooled for testing. Females were processed individually or grouped in pools of 2–15 specimens. Legs were removed from each collected sample and stored in RNAlater at – 80 °C for subsequent individual analysis of positive pools.

RNA extraction was performed with the Quick RNA Viral Kit (Zymo Research, Irvine, CA, USA; Ref: 1035) following the manufacturer's instructions with a final elution volume of 50 μl. In extractions of individual females the elution volume was 25 μl. For blood-meal analysis we only used fully engorged (100% blood fed) and partially engorged (around 75% blood fed) females [34]. *Aedes aegypti* females were processed individually, and RNA/DNA extraction was done using the Quick DNA/RNA Viral kit (Zymo Research; Ref: D7021) to perform virus and blood-meal source analysis.

### Multiplex real-time reverse transcription-PCR assays

For detection of ZIKV, DENV and CHIKV RNA, we used the ZDC Multiplex RT-PCR Assay (Bio-Rad, Hercules, CA, USA) according to the manufacturer’s instructions. The multiplex RT-PCR mix contained 5 μl of the extracted RNA and the following reagents: 4.9 μl of nuclease-free water, 12.5 μl of 2× iTaq™ Universal Probes One-Step Reaction Mix, 0.6 μl of iScript™ Reverse transcriptase and 2 μl of ZDC Multiplex RT-PCR Assay Mix, to a final reaction volume of 25 μl. Reactions were run in 96-well plates using an ABI 7500 FAST Dx thermocycler (Applied Biosystems®, Thermo Fisher Scientific, Waltham, MA, USA). The ZDC kit includes a lyophilized positive control for ZIKV, DENV and CHIKV and an internal RNA-negative control. We also used purified DENV and ZIKV samples from cell cultures stored in the sample bank of the Centro de Investigaciones en Microbiología y Parasitología Tropical (CIMPAT), as additional positive controls. Controls were processed simultaneously with the samples and following the same methods. The standard PCR cycling method was selected, and the fluorescence capture was set to detect emissions through the following channels: FAM for ZIKV, HEX for CHIKV, Texas Red for DENV and CY5 for the internal control. Thermocycling parameters were: reverse transcription (RT) at 50 °C, 15 min; then denaturation at 94 °C/2 min; followed by fluorescence detection at 94 °C/15 s, 55 °C/40 s, 68 °C/30 s for 45 cycles. Amplification curves were evaluated by each probe, and the software’s default threshold line was placed above the background signal. Amplification curves with Cq values ≥ 37 were considered negative. In samples positive for CHIKV, the same procedure was performed using the legs of each mosquito.

### DENV serotyping

In pools positive for DENV, serotyping was performed using the legs of each mosquito. In one pool, it was not possible to determine the serotype using the legs, so serotyping was performed with RNA extracted from the pool.

Samples were analyzed for DENV serotype determination (serotype 1 [DENV-1], serotype 2 [DENV-2], serotype 3 [DENV-3], serotype 4 [DENV-4]) through RT-PCR. Reactions were performed using SuperScript III Platinum One-Step Quantitative RT-PCR System (Thermo Fisher Scientific). To assemble a multiplex RT-PCR reaction, an aliquot of total RNA (1 pg to 1 µg) was mixed with the following reagents: 25 µl of 2× reaction mix, 10 µM of forward and reverse primers (DENV-1, DENV-2, DENV-3 and DENV-4), 10 µM of each Taqman probe [36] (Additional file 1: Table S1) and 1 µl of SuperScript III RT/Platinum Taq mix, to a final reaction volume of 50 µl. We used RNA extracted from viral culture supernatants from each of the four DENV serotypes as positive control, and RNA extracted from legs of non-infected mosquitoes as negative control.

Individual reactions were placed in 96-well plates and run using an ABI 7500 Fast thermocycler (Applied Biosystems®, Thermo Fisher Scientific). The standard cycling method was selected and fluorescence capture was set to detect emissions through the FAM, VIC, NED, and JOE channels in each well. Thermocycling parameters were: RT at 50 °C, 15 s; RT inactivation at 95 °C/2 min; fluorescence detection at 95 °C/15 s for 40 cycles; and annealing at 60 °C/30 s. Amplification curves were evaluated by serotype against the software’s default threshold line. Amplification curves with Ct values of ≥ 37 were considered negative.

### Blood-meal analysis

Fully and partially engorged females were processed for blood-meal analysis. DNA templates were tested with primers for human beta-globin gene [37] and mammalian C primers [38]. For beta-globin PCR, 5 μl total DNA was mixed with the following reagents: 12.5 μl of 2× GoTaq Green Master Mix, 10 μM of forward and reverse primers (Additional file 1: Table S1), to a final reaction volume of 25 μl. The thermocycling conditions consisted of 1 cycle at 95 °C, 5 min; then 95 °C/1 min, 58 °C/1 min, 72 °C/1 min for 35 cycles; with a final cycle of 72 °C/10 min; the size of the specific PCR product was 110 bp. Some samples that were positive for human beta-globin were purified, sequenced and identified by comparing the DNA sequence to GenBank databases using BLAST (National Center for Biotechnology Information [NCBI]). Sequences with an elevated identification percentage (≥ 97%) were accepted as positive. For the PCR performed with primers for mammals, 5 μl total DNA was mixed with the following reagents: 12.5 μl of 2× GoTaq Green Master Mix, 10 μM of forward and reverse primers (Additional file 1: Table S1), to a final reaction volume of 25 μl. The thermocycling conditions consisted of 1 cycle at 95 °C, 5 min; then 95 °C/1 min, 55 °C/1 min, 72 °C/1 min for 32 cycles; the size of the specific PCR product was 395 bp.

Sequencing was performed for samples that did not amplify for human-beta globin. For the analyses with mammalian C primers, DNA extracted from dog blood was used as a positive control and DNA extracted from chicken blood was used as a negative control. For human beta-globin molecular assays, DNA extracted from human blood was used as a positive control and DNA extracted from dog blood was used as a negative control. It was considered that a mosquito had fed on human blood if amplification was positive for human beta-globin and/or *Homo sapiens* sequence was obtained after amplification with mammalian primers.

### Epidemiological surveillance

Records of reported human cases of dengue, chikungunya and Zika for Ibagué were obtained from the Sistema Nacional de Vigilancia en Salud Pública (SIVIGILA) [13].

## Results

### Mosquito abundance

**Fig. 2:**
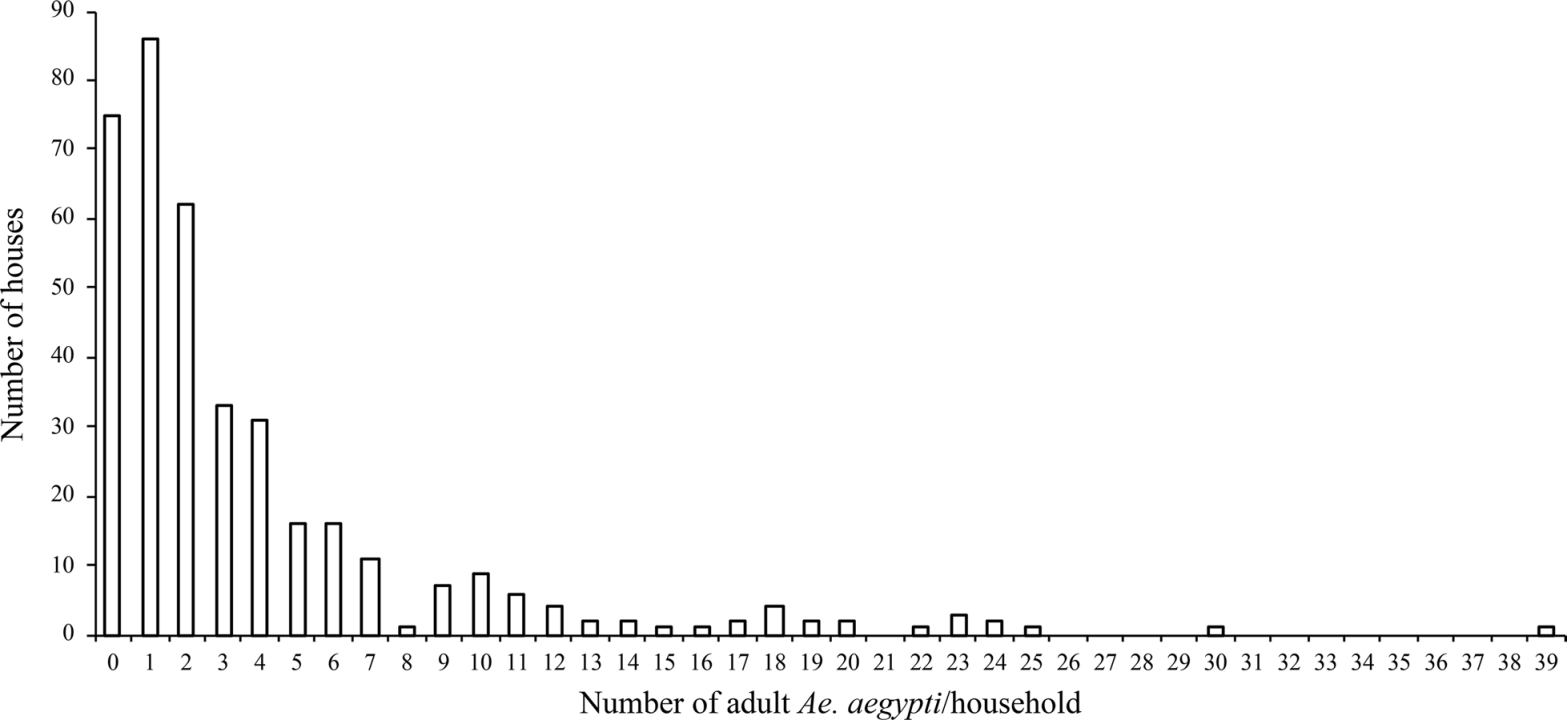
Number of houses in which the corresponding number of adult *Aedes aegypti* was collected

In total, 1463 *Ae. aegypti* adult mosquitoes were collected (Additional file 2: Table S2). In addition, we collected seven *Ae. albopictus* individuals, representing the first report for this species in Ibagué. Due to the low number of collected *Ae. albopictus* individuals, our data analyses concentrated on *Ae. aegypti*, unless otherwise specified. The average *Ae. aegypti* abundance (mean ± standard deviation; [SD]) was 3.8 ± 5.3 mosquitoes/household, and more females than males were collected: 2.1 ± 2.9 females/household vs. 1.7 ± 2.9 males/household (Friedman test:* Q* = 12.44, *df* = 1, *P* < 0.001). In total, 307 houses (80.4%) were positive for *Ae. aegypti*, and there was a large variation in the number of mosquitoes that was captured per household, as indicated by a coefficient of variation (CV) ≥ 1 (CV = SD/mean = 1.4) (Fig. 2).

In total, 1182 *Ae. aegypti* mosquitoes were collected using the aspirator, 243 with the BG-Sentinel trap, 25 with the resting trap and 13 with the CDC trap. Of these, 605 females (42.3% were fed) were collected with the aspirator and 170 females (8.2% were fed) with BG-Sentinel traps.

### Intradomicile and peridomicile spaces

**Fig. 3:**
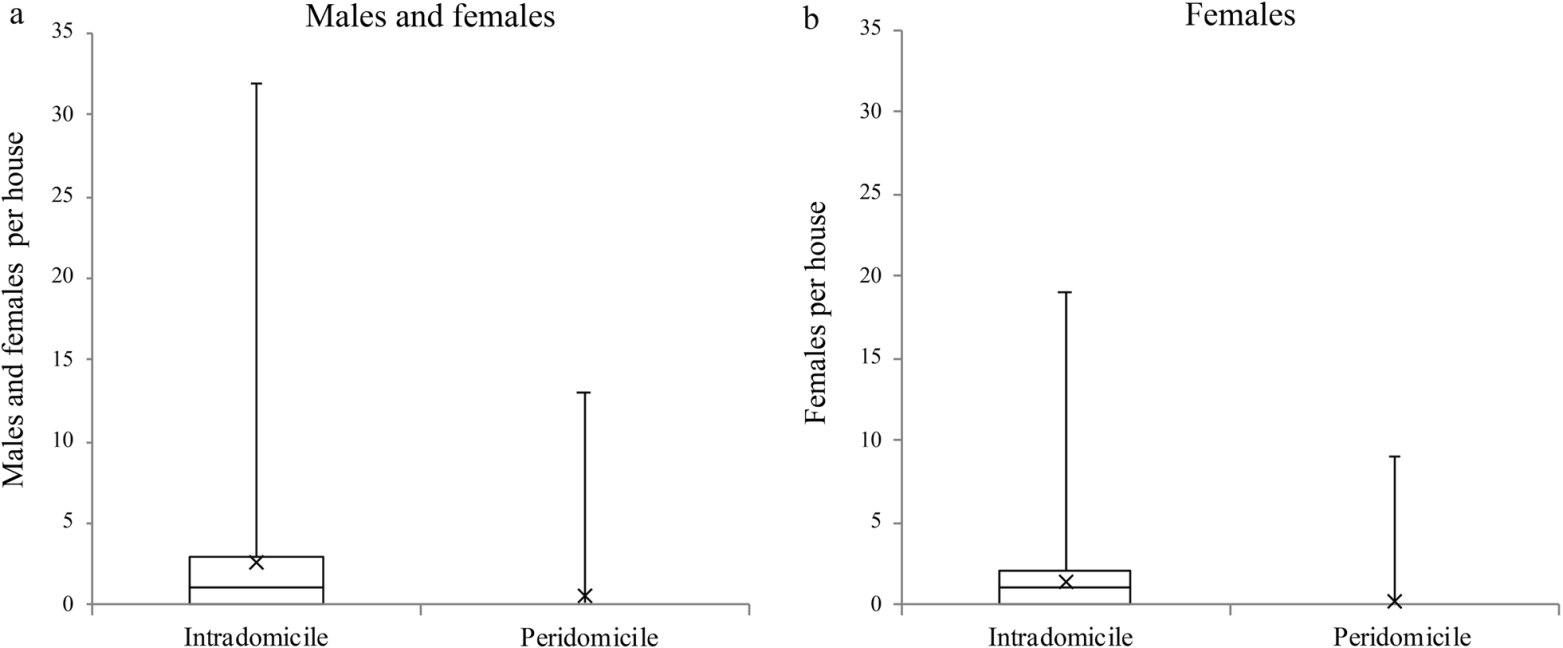
*Aedes aegypti* adults collected per house in the intradomicile and peridomicile spaces. **a** Number of males and females collected per house, **b** number of females collected per house. The box plot shows the median and interquartile range (IQR), mean (x) and maximum (upper bar) values for each category

More mosquitoes were collected using the aspirator in the intradomicile space than in the peridomicile space (*Q* = 106.67, *df* = 1, *P* < 0.0001), and additionally, more females (*Q* = 138.19, *df* = 1, *P* < 0.0001) and blood-fed females (*Q* = 98.84, *df* = 1, *P* < 0.0001) were collected in the intradomicile space (Fig. 3).

In total, 90 breeding sites positive for *Ae. aegypti* and/or *Ae. albopictus* were found in 75 houses, and in most of these (90%) only *Ae. aegypti* was found. In the rest of the breeding sites, the following species were identified: 1.1% only *Ae. albopictus*, 5.6% *Ae. aegypti* and *Ae. albopictus*, 2.2% *Ae. aegypti* and *Culex* spp. and 1.1% *Ae. albopictus* and *Wyeomyia* spp. A higher number of *Ae. aegypti*-positive breeding sites were found in the peridomicile space than in the intradomicile space (76% in the peridomicile space vs. 24% in the intradomicile; Fisher’s exact test: *P* < 0.001). Most breeding sites corresponded to open top watertanks mainly used for laundry (37.5%) (known in Colombia as “albercas”), followed by aquatic plant pots, flower vases or plant pot base plates (26.1%) and buckets (9.1%). The rest of the breeding sites were found in a diverse range of containers, including decorative fountains, bottles and animal feeders.

A logistic regression analysis to assess the relationship between the number of *Ae. aegypti* adults and the presence of *Ae. aegypti* breeding sites per house showed no statistically significant association (logistic regression:* R*^2^ = 0.0018, *df* = 1, *P* = 0.4).

### Low and high SES neighborhoods

**Fig. 4:**
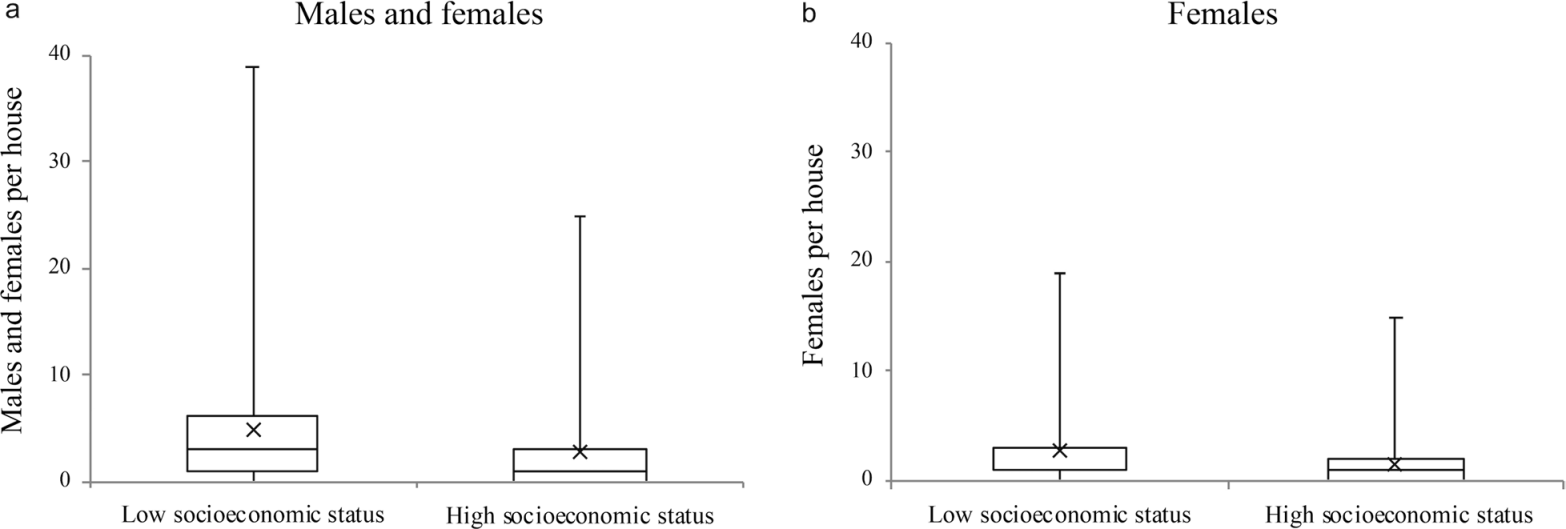
*Aedes aegypti* collected per house in low and high SES neighborhoods. **a** Number of males collected per house, **b** number of females collected per house. The box plot shows the median and IQR, mean (x) and maximum (upper bar) values for each category

Mosquitoes were collected in all sampled neighborhoods. However, more *Ae. aegypti* mosquitoes (males and females) were collected in low than in high SES neighborhoods (Mann–Whitney test:* U* = 12,318, *df* = 1, *P* < 0.0001). Analyses performed only with females (*U* = 13,087, *df* = 1, *P* < 0.0001) and with blood-fed females (*U* = 12,140, *df* = 1, *P* < 0.0001) also showed a higher abundance of these subgroups in low SES neighborhoods (Fig. 4). We did not find significant differences between low and high SES neighborhoods according to the number of households positive for *Ae. aegypti* breeding sites (Fisher’s exact test: *P* = 0.07).

Characteristics of high and low SES neighborhoods are shown in Table 1 and in Additional file 2: Table S3. No significant differences were found in terms of access to public services or in construction type, as every house situated in a high SES neighborhood had access to electricity, water and sewer service; in low SES neighborhoods, every house had a connection to water and sewer service, and all but three houses had access to electricity. In relation to construction type, all houses in high SES neighborhoods were solid constructions, while in low SES neighborhoods, 99.4% of the houses had permanent ceilings and solid walls, and 97.9% had permanent floors (Table 1). No differences were found in the number of inhabitants per house, nor in the percentage of families that owned a computer, motorcycle, television or fridge (Table 1). Significant differences were found in relation to the percentage of households that had air conditioning and a car. In high SES neighborhoods, 43.9% of the households had air conditioning and 95.7% owned a car, while in contrast, none of the households from low SES neighborhoods had air conditioning, and 24.5% owned a car (Table 1). An investigation of associations between household characteristics and mosquito abundance will be presented separately in a forthcoming publication (Talbot et al. in preparation).

**Table 1:** Characteristics of households in low and high socioeconomic status neighborhoods

Characteristics	Low SES household (*n* = 192)	High SES household (*n* = 187)
Mean number of inhabitants	3.8	3.4
Electricity service	98.4%	100%
Water and sewer service	100%	100%
Permanent ceiling	99.4%	100%
Solid walls	99.4%	100%
Permanent floor	97.9%	100%
Mean number of years family has lived in the house	22.1	9
Percentage of families owning house	74.5%	89.8%
Percentage of households with TV	98.9%	100%
Percentage of households with fridge	99.4%	100%
Percentage of households with air conditioning^a^	0%	43.9%
Percentage of households with computer	69.8%	94.6%
Percentage of households with motorcycle	41.1%	12.8%
Percentage of households with car^a^	24.5%	95.7%

SES, Socioeconomic status

^a^The percentage of houses with air conditioning and the percentage of households with a car were significantly different between high and low SES households based on Bonferroni-adjusted α-value (0.05/number of null hypotheses = 0.05/14 = 0.0035)

### Variation in mosquito abundance over time

**Fig. 5:**
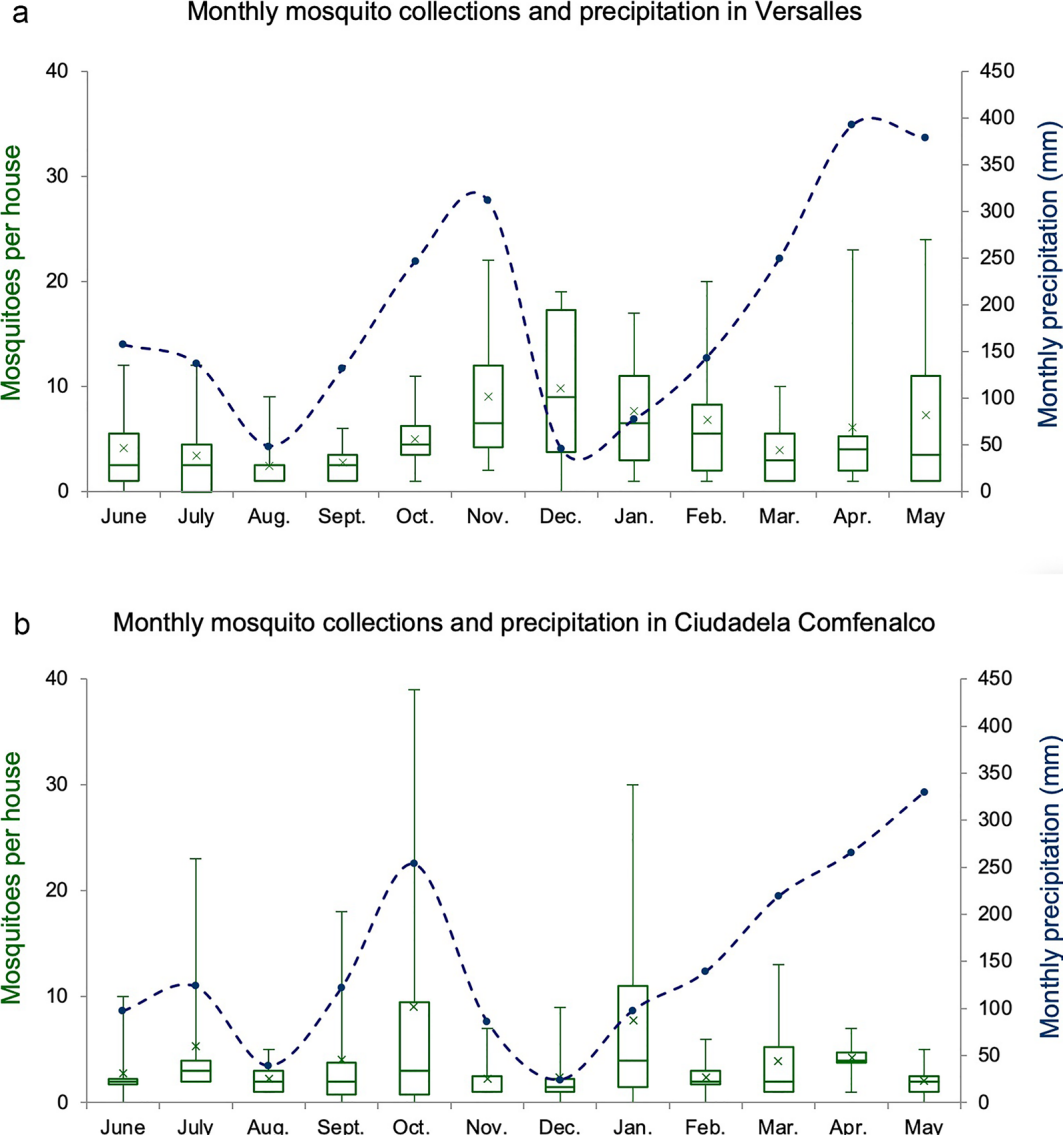
*Aedes aegypti* collected monthly and monthly precipitation between June 2018 and May 2019 in two low SES neighborhoods: **a** Versalles, **b** Ciudadela Comfenalco. The box plot shows the median and IQR, mean (x) and maximum (upper bar) and minimum values (lower bar) values for each category

Our analysis of mosquito abundance over time was restricted to the two low SES neighborhoods, namely Versalles and in Ciudadela Comfenalco, where sampling was performed every month during the study period. Monthly *Ae. aegypti* abundance in Versalles showed a slight bi-modal pattern with peaks in November–January and April–June, while in Ciudadela Comfenalco there was no observable pattern (Fig. 5). There were no significant differences in monthly mosquito abundance in either neighborhood (Versalles: Kruskal–Wallis test:* H* = 15.79, *df* = 11, *P* = 0.139; Ciudadela Comfenalco:* H * = 9.63, *df* = 11, *P* = 0.53) (Fig. 5). In addition, no differences were found in the number of houses positive for presence of breeding sites per month (Versalles:* H* = 6.24, *df* = 11, *P* = 0.333; Ciudadela Comfenalco:* H* =  8.22, *df* = 11, *P* = 0.55).

Mosquito abundance was not significantly correlated with temperature (Spearman correlation: Versalles, *r*_*s*_ = 0.020, *P* = 0.79; Ciudadela Comfenalco, *r*_*s*_ = 0.07, *P* = 0.85), humidity (Versalles, *r*_*s*_ = 4.80E-5, *P* = 0.99; Ciudadela Comfenalco, *r*_*s*_ = 5.2E-6, *P* = 0.88) or precipitation (Versalles, *r*_*s*_ = 0.015, *P* = 0.41; Ciudadela Comfenalco, *r*_*s*_ = 0.082, *P* = 0.091). Similarly, these three variables did not show significant associations with breeding site presence (temperature: Versalles,* R*^2^ = 0.001, *df* = 1, *P* = 0.74; Ciudadela Comfenalco,* R*^2^ = 0.002, *df* = 1, *P* = 0.6; humidity: Versalles,* R*^2^ = 0.0009, *df* = 1, *P* = 0.95; Ciudadela Comfenalco,* R*^2^ = 0.001, *df* = 1, *P* = 0.8; precipitation: Versalles,* R*^2^ = 0.0031, *df* = 1, *P* = 0.62; Ciudadela Comfenalco,* R*^2^ = 0.0071, *df* = 1, *P* = 0.1).

### Virus detection assays

Virus detection assays were performed on 799 *Ae. aegypti* females (482 females grouped in 133 pools and 317 individual females) and seven *Ae. albopictus* individual females (Additional file 2: Table S4). In total, four *Ae. aegypti* pools were positive for DENV and one *Ae. aegypti* pool was positive for CHIKV. ZIKV was not detected in any tested sample. Individual females were not positive for any virus. After serotyping, DENV-1 was identified. All positive mosquitoes were collected in low SES neighborhoods. Most of the infected females were collected in the intradomicile space (Table 2). Positive samples and the respective positive controls had amplification curves with Cq values < 37, contrary to negative samples and the negative control that had Cq values  ≥ 37.

**Table 2:** *Aedes aegypti* mosquitoes positive for arboviruses by neighborhood and location of collection

Neighborhood	Month	Mosquitoes per pool (*n*)	ZIKV	DENV^a^	CHIKV	No. of positive individuals per pool^b^	Household space
Versalles	November 2018	2	–	+ (DENV-1)	–	1	Intradomicile
Versalles	December 2018	2	–	–	+	Undetermined	Intradomicile
Versalles	February 2019	13	–	+ (DENV-1)	–	2	Intradomicile
Versalles	March 2019	3	–	+ (DENV-1)	–	Undetermined	Intradomicile
Ciudadela Comfenalco	March 2019	6	–	+ (DENV-1)	–	2	Peridomicile

DENV, Dengue virus, CHIKV, chikungunya virus; ZIKV, Zika virus

^a^DENV-1, Dengue virus serotype 1

^b^Reverse transcription-quantitative PCR was performed on extractions of the legs of each individual that made up the pool

The percentage of positive pools was 3% for DENV and 0.8% for CHIKV. The minimum infection rate (MIR) was calculated as the number of positive pools/number of processed individuals × 1000 [39], resulting in a MIR of 8.3 for DENV (4 positive pools/482 total processed individual mosquitoes × 1000) and a MIR of 2.1 for CHIKV (1 positive pool/482 total processed individual mosquitoes × 1000).

### Blood-feeding preferences

A total 148 females were processed for the analysis of blood meals. Human blood was found in 77.7% of the processed samples, with no amplification or inconclusive results in the remaining 22.3% of samples. In the PCR that was run with human beta-globin primers, positive samples and the positive control amplified at 110 bp. The size of the amplicon obtained with mammalian primers in positive samples and the positive control corresponded to 395 bp. In negative samples and the corresponding negative control, no amplification was obtained.

### Epidemiological surveillance

**Fig. 6:**
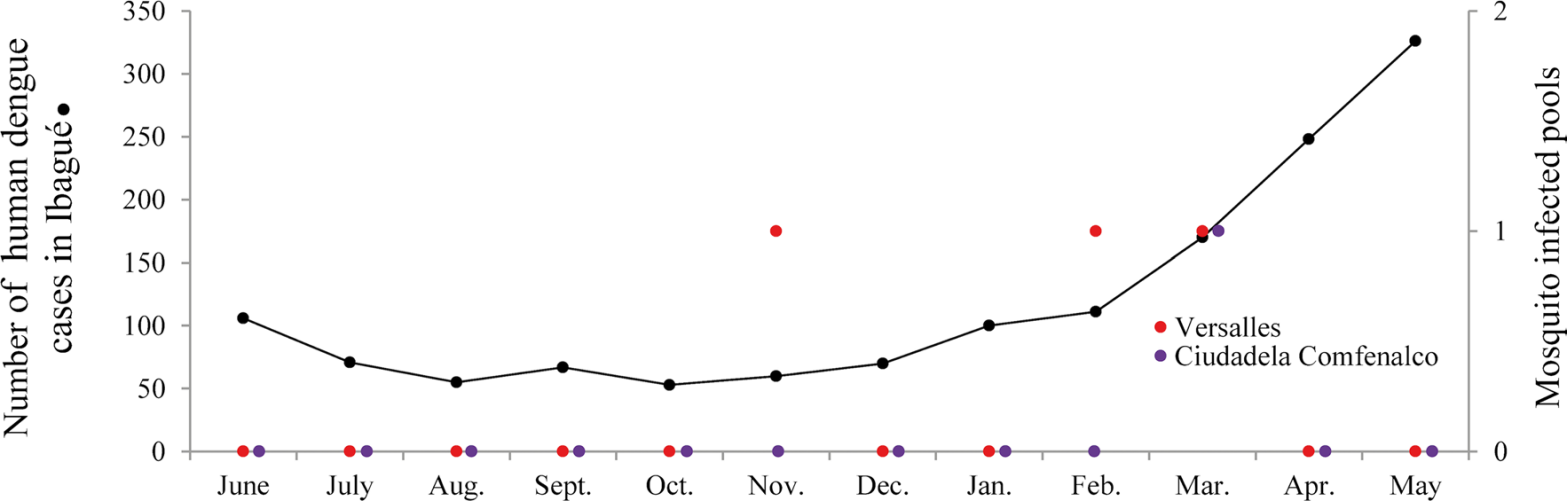
Number of human dengue cases in Ibagué and number of *Aedes aegypti*-infected pools between June 2018 and May 2019

In Ibagué, a typical number of dengue cases was reported during the initial period of mosquito collections; however, from late January until late February 2019, the Health Department recorded an increase in the number of cases followed by an epidemic that extended until March 2020 (Fig. 6).

## Discussion

In our study, DENV was detected in mosquitoes as early as November 2018, slightly preceding the increase in number of reported human cases, while multiple DENV-positive mosquito pools were detected in early 2019, coinciding with further increases in human dengue incidence [40–45].

This study documents findings of the co-circulation of DENV and CHIKV in *Ae. aegypti* females in Ibagué, Colombia. The percentage of positive pools was 3% for DENV and 0.8% for CHIKV. Our results are within the ranges obtained in other studies performed in Latin America. In these studies, infection rates of between zero and 16.2% were detected for DENV [46, 47] and between zero and 12.5% for CHIKV [48, 49].

While we only detected the DENV-1 serotype, circulation of DENV-2 and DENV-3 in Tolima was also registered in 2018 and 2019 in human samples [50, 51]. DENV-1 and DENV-2 are currently the most commonly detected serotypes in Colombia [10, 52], and DENV-4 has also been detected sporadically [53, 54].

Our detection of CHIKV in only one mosquito pool may reflect the marked decline in virus transmission following the epidemic period that occurred after the introduction of the virus in 2014, decreasing from 1359 cases per 100,000 inhabitants in 2015 to two cases per 100,000 inhabitants in 2019 [8, 55]. A similar trend in transmission was observed for ZIKV, which was not detected in our study, wherein an epidemic was observed following the introduction of the virus, followed by a steep decline in the number of Zika cases, from 377.7 cases per 100,000 inhabitants during the epidemic period in 2015 and 2016 to 1.6 cases per 100,000 inhabitants in 2019 [8, 18]. The decreasing tendency in the number of chikungunya and Zika cases after the acute epidemic period is mainly attributed to the development of immunity in humans after infection by these viruses, which reduces both the number of infected people and infected mosquitoes [56, 57].

Our results on co-circulation of DENV and CHIKV viruses in sampled mosquitoes within the same neighborhood are supported by findings from prior studies that detected co-circulation within the same city [28] or from nearby municipalities [29]. To our knowledge, no co-circulation of ZIKV with DENV and/or CHIKV has been identified in naturally infected mosquitoes within the same city [29, 46–49].

Our results on the detection of infected mosquitoes exclusively in low SES neighborhoods, which also harbor higher total mosquito and blood-fed female mosquito abundance, is supported by the literature on the subject, suggesting that DENV may be closely associated with poverty [58]. However, the relationship between risk of DENV infection and disease and poverty is currently not well understood [59]. On the global scale, several factors have been suggested as drivers of disease transmission, including rapid urbanization and globalization, with its associated global increase in travel and trade. On the local scale, lack of effective mosquito control, water management practices, human migration on a local scale and the introduction of new and more virulent dengue serotypes into an immunologically naive population have been identified as important drivers of disease transmission [12, 60, 61]. Specifically, Ibagué is characterized by having high population growth, inadequate water storage practices, high density of water storage containers and elevated precipitation, all of which contribute to a high incidence of dengue [62].

Our results on the anthropophagic behavior of *Ae. aegypti* are supported by findings from previous studies [63–66]. Our results on higher abundance of total adult, total female and blood-fed female mosquitoes in the intradomicile versus the peridomicile space are also supported by findings from previous studies [64, 65, 67]. These findings, together with our result showing an association of infected females with the intradomicile space, clearly demonstrate the direct risk these mosquitoes pose to humans through disease transmission. On the other hand, our results showing a higher number of breeding sites in the peridomicile space suggest that oviposition takes place mostly outside the dwelling and that mosquitoes come indoors to feed, mate and digest blood, indicating that interventions should be addressed towards measures that reduce the entrance of mosquitoes indoors, such as using screens in windows and doors, keeping doors and windows closed and implementing breeding site control in the peridomicile.

Our results suggest that mosquito abundance and presence of breeding sites were not related with ambient temperature and humidity. This may be due to the location of Colombia on the Equator, which leads to low thermal variation throughout the year. This is particularly important in Ibagué, where the thermal variation is ≤ 2 °C year-round [68]. However, monthly precipitation varies greatly throughout the year. Ibagué has an annual precipitation of approximately 1691 mm, which is concentrated in two rainy periods: April–May and October [68]. Mosquito breeding sites in Colombia are usually detected in artificial containers, such as tanks, buckets, flower vases, animal feeders, bottles and water fountains, that are mainly filled with water by humans [12]. In our study site, most containers associated with breeding sites were filled by house-owners in order to store water (open water tanks and buckets) or to keep ornamental plants (aquatic plant pots, flower vases or plant pot base plates). Therefore, mosquito breeding sites may be mostly managed by people. This highlights the importance of the role of the community on breeding site control and gives insights into the importance of education and outreach activities in the communities [69, 70].

In our study, the mosquito collection method that yielded the highest number of captured *Ae. aegypti* mosquitoes was the Prokopack aspirator, followed by the BG-Sentinel trap. Few *Ae. aegypti* mosquitoes were captured using the resting and CDC light traps. Several studies have shown the efficiency of the Prokopack aspirator [71–74], the Back-Pack aspirator [75] and the BG-Sentinel trap for collecting *Ae. aegypti* mosquitoes [76, 77]*.* As in our study, BG-Sentinel traps collect a higher number of *Ae. aegypti* compared to CDC light traps [78]. We did not find comparative studies between the Prokopack aspirator and the BG-Sentinel trap. However, studies have been performed using both the BG-Sentinel trap and the Back-Pack aspirator. Maciel-de-Freitas et al. [79] and Williams et al. [80] found that the BG-Sentinel trap was overall more efficient than the Back-Pack aspirator at collecting *Ae. aegypti*. As shown in our study, Sivagnaname and Gunasekaran [81] reported BG-Sentinel traps collected mostly unfed *Ae. aegypti* female mosquitoes and Williams et al. [80] found aspirators collected proportionally more blood-fed *Ae. aegypti* female mosquitoes.

Our study provides the basis for the first report of *Ae. albopictus* in the city of Ibagué. This highly invasive species was found for the first time in Colombia in 1998 [22] and since then it has spread to 15 out of 32 departments of the country [23–27]. As this mosquito is capable of transmitting DENV, CHIKV and ZIKV [82–84], it is of importance to health municipal authorities. Targeted surveillance activities for *Ae. albopictus* in the municipality should be conducted to evaluate the risk it poses to the local population.

## Conclusions

This research constitutes the first comprehensive entomological characterization of *Aedes* spp. in Ibagué, Colombia, confirming the co-circulation of DENV (serotype 1) and CHIKV in *Ae. aegypti* females. Interestingly, 3 years after its introduction in Colombia, ZIKV was not detected in any collected mosquitoes. The positivity for DENV and CHIKV, predominance of mosquitoes in the intradomicile space and the high proportion of females that had fed on humans highlight the high risk for arbovirus transmission in Ibagué, but may also provide the opportunity for establishing effective control strategies. Housing improvements to reduce mosquito entry and community participation for the elimination and control of peridomestic breeding sites may be important elements to reduce arbovirus transmission in this and other Colombian cities.

## Supplementary Information

**Additional file 1:Table S1.** Sequences of probes and primers.

**Additional file 2:Table S2.** Data on adult mosquitoes collected in Ibagué, Colombia. **Table S3.** Household questionnaire data, Ibagué, Colombia. **Table S4.** Mosquito molecular data, Ibagué, Colombia.

## Data Availability

Data supporting the conclusions of this article are included within the article and its Additional files 1 and 2, which are also available at Figshare. 10.6084/m9.figshare.15067221 and 10.6084/m9.figshare.15067839.

## Abbreviations

CHIKV: Chikungunya virus
CV: Coefficient of variation
DENV: Dengue virus
DENV-1: Dengue virus serotype 1
DENV-2: Dengue virus serotype 2
DENV-3: Dengue virus serotype 3
DENV-4: Dengue virus serotype 4
MIR: Minimum infection rate
RT: Reverse transcription
SES: Socioeconomic status
ZIKV: Zika virus
