# Supplementary material for: Entomological characterization of Aedes mosquitoes and arbovirus detection in Ibagué, a Colombian city with co-circulation of Zika, dengue and chikungunya viruses
Source: Parasit Vectors. 2021 Sep 6;14:446. doi: 10.1186/s13071-021-04908-x (PMC8419972; doi:10.1186/s13071-021-04908-x)
Supplement: Supplementary file 1 — Additional file 1:Table S1. Sequences of probes and primers. [file 13071_2021_4908_MOESM1_ESM.docx]

**Additional file 1: Table S1.** Sequences of probes and primers.

| **ID** | **Sequence** | **Reference** |
| --- | --- | --- |
| D1-Probe | FAM-CATGTGGYTGGGAGCRCGC-BHQ1 | [1] |
| D2-Probe | NED-CTCYCCRAGAACGGGCCTCGACTTCAA-BHQ1 |  |
| D3-Probe | VIC-ACCTGGATGTCGGCTGAAGGAGCTTG- BHQ2 |  |
| D4-Probe | JOE-TYCCTACYCCTACGCATCGCATTCCG-BHQ3 |  |
| D1-F | CAAAAGGAAGTCGYGCAATA |  |
| D1-R | D1-R CTGAGTGAATTCTCTCTACTGAAC |  |
| D2-F | D2-F CAGGTTATGGCACTGTCACGAT |  |
| D2-R | D2-R CCATCTGCAGCAACACCATCTC |  |
| D3-F | D3-F GGACTGGACACACGCACTCA |  |
| D3-R | D3-R CATGTCTCTACCTTCTCGACTTGTCT |  |
| D4-F | D4-F TTGTCCTAATGATGCTGGTCG |  |
| D4-R | D4-R TCCACCTGAGACTCCTTCCA |  |
| MAM-F | CCATCCAACATCTCAGCATGATGAAA | [2] |
| MAM-R | GCCCCTCAGAATGATATTTGTCCTCA |  |
| PCO3 | ACACA ACTGT GTTCA CTAGC | [3] |
| PCO4 | CAACT TCATCCACGT TCACC |  |

**References**

1. Santiago GA, Vergne E, Quiles Y, Cosme J, Vazquez J, Medina JF, et al. Analytical and clinical performance of the CDC real time RT-PCR assay for detection and typing of dengue virus. PLoS Negl Trop Dis. 2013.7:e2311.
2. Molaei M, Andreadis TG, Armstrong PM, Andreson JF, Vossbrinck C. Host feeding patterns of *Culex* mosquitoes and West Nile virus transmission, northeastern United States. Emerg Infect Dis. 2006;12:468-74.
3. Jalal S, Malekshahi S, Ghalejoogh ZY, Ghavvami N, Jandaghi N, Shahsiah R, et al. Detection and typing of human papilloma viruses by nested multiplex polymerase chain reaction assay in cervical cancer. Jundishapur Microbiol. 2015;8:1-5.
